# Changing patterns in the burden of paediatric injuries during the COVID-19 pandemic: a study in Mozambique’s central hospitals

**DOI:** 10.1186/s12913-023-10073-x

**Published:** 2023-10-06

**Authors:** Vanda Amado, Sebastien Trott, Jette Möller, Maria Tereza Couto, Lee Wallis, Lucie Laflamme

**Affiliations:** 1 Present Address: Department of Global Public Health, Karolinska Institutet, Widerströmska Huset, Tomtebodavägen 18A, 171 77 Stockholm, Sweden; 2 Faculty of Medicine, Eduardo Mondlane University, Maputo, Mozambique; 3 Maputo Central Hospital, Maputo, Mozambique; 4 Department of Emergency Medicine, Warren Alpert Medical School of Brown University, Providence, RI USA; 5 Mozambique Medical Council Maputo, Maputo, Mozambique; 6 Division of Emergency Medicine, Faculty of Health Sciences, University of Cape Town, Cape Town, South Africa; 7 Institute for Social and Health Sciences, University of South Africa, Pretoria, South Africa

**Keywords:** Sub-Saharan Africa, Mozambique, LMIC, Trauma care, Paediatric injuries, COVID-19

## Abstract

**Introduction:**

There is a substantial body of knowledge on the effects of the COVID-19 pandemic on injuries showing frequent but inconsistent reductions in both volume and pattern. Yet, studies specifically addressing children are less common, not least from low- and middle-income countries. This study investigated whether changes in the pattern and outcome of paediatric injury admissions to Mozambique’s four regional referral hospitals during 2020.

**Methods:**

Clinical charts of paediatric patients presenting to the targeted hospitals with acute injuries were reviewed using a set of child, injury, and outcome characteristics during each of two consecutive restriction periods in 2020 using as a comparator the same periods in 2019, the year before the pandemic. Differences between 2020 and 2019 proportions for any characteristic were examined using the t-test (significance level 0.05).

**Results:**

During both restriction periods, compared with the previous year, reductions in the number of injuries were noticed in nearly all aspects investigated, albeit more remarkably during the first restriction period, in particular, greater proportions of injuries in the home setting and from burns (7.2% and 11.5% respectively) and a reduced one of discharged patients (by 2.5%).

**Conclusion:**

During the restrictions implemented to contend the pandemic in Mozambique in 2020, although each restriction period saw a drop in the volume of injury admissions at central hospitals, the pattern of child, injury and outcome characteristics did not change much, except for an excess of home and burn injuries in the first, more restrictive period. Whether this reflects the nature of the restrictions only or, rather, other mechanisms that came into play, individual or health systems related, remains to be determined.

## Background

Although a preventable cause of mortality and morbidity, injuries remain a leading cause of death and long-term disability worldwide, particularly for children [1, 2]. In 2017, the latest data available, they contributed 10.1% (9.7%–10.5%) to the global burden of disease [3]. Given that for each child injury death, about 12 others are either hospitalized or suffer from permanent disability, the health system implications of childhood injuries are immense [1]. Currently, over 95% of injury deaths occur in low- and middle-income countries (LMICs) [3, 4], where the means to prevent them from occurring and minimise their sequelae are often limited [5–8]. In that respect, compared with high-income countries (HICs), LMICs tend to have less established emergency care systems, including prehospital, emergency, anaesthesia and surgery, and rehabilitation services [8, 9]. Limitations in prehospital care in particular lead to later presentations, more challenging resuscitations, and worse outcomes [9]. Such issues can be exacerbated in periods when health systems are under exceptional pressure, like during pandemics [10], as witnessed around the world through the COVID-19 pandemic [11].

In Sub-Saharan Africa (SSA), the context of the current study, the first COVID case was reported in Nigeria on January 28, 2020, [12], while the virus spread throughout the region, including Mozambique, where the first case was reported on March 2 [13]. The pandemic was an additional challenge for the region, which faced shortages of medical supplies, human resources, and personal protective equipment, and lacked both a reliable electricity supply and a functioning supply chain. To contain the spread of the virus and minimize the burden on overcrowded health systems, the governments of several SSA countries began implementing social and physical distancing measures e.g., movement and travel restrictions, stay-at-home policies, and lockdowns. COVID-19 awareness campaigns were launched alongside the promotion of handwashing. Countries that had never produced surgical masks, gloves, sanitisers, or ventilators engaged in domestic production. African countries created the Africa Taskforce on Coronavirus Preparedness and Response (AFTCOR), a continent-wide effort engaging in a range of public health activities e.g., monitoring systems; laboratory diagnosis and subtyping, surveillance; infection prevention and control in health care facilities; clinical treatment of people with severe COVID-19; risk communication; and supply chain management and stockpiles [12, 14]. In Mozambique [13, 15] a scientific committee was established to guide and advise the government’s response, a surveillance system for COVID-19 infection was put into place, and containment measures were enacted too. These measures – and the pandemic itself – had significant impacts on national health systems.

When it comes to child injuries, the body of knowledge on the effect of the COVID-19 pandemic has been synthesized in several age-specific or all-ages reviews concerning injuries in general [16], road traffic injuries (RTIs) [17, 18], falls and other orthopaedics injuries [19], burns [20], and violence-related injuries [21, 22], each covering around 25–48 studies depending on the subject. A common observation among those reviews is that volume of injuries tended to go down, at least following the implementation of the first restrictions, and that injury severity seen at hospital tended to increase. However, this pattern was not consistent across outcomes or settings, or across time within settings. This is reflected, for instance, in studies about injuries in general where paediatric injuries presenting to emergency departments (ED) were seen to decrease but there were increases in ward admissions, and no changes in intensive care unit (ICU) admissions [16]. Further, it has been observed that those overall reductions resulted often from considerable reductions in RTIs [16–19] and, to some extent, in sport and leisure related injuries [19]. The most common explanation for lowered numbers is that of reduced hazard exposure as a consequence of confinement and mobility restrictions [16, 19, 23, 24]. While the explanation is very plausible [16, 24], it cannot help to explain a rise in the volume of injuries during the same restriction period shown in a few studies [18]. Here, we may be seeing the result of poor enforcement of restrictions, or a change in the psychological impact and fear of contamination initially experienced at the population level that kept people away from the clinical setting, including children's guardians [16, 25].

As indicated above, restriction periods could also be characterized by shifts in injury patterns [18, 19, 23], reflecting changes in exposure. Most typically, while RTIs went down, confinement measures were associated with an increase in the number (or proportion) of burns [26–28] and violence-related injuries [21, 22, 29, 30]. Most typically, burns became more frequent and delays and reductions were reported in hospital paediatric burns management [20, 26–28]. For violence against children, abused-related injuries rose in hospital-based care but decreased in police and child protective services [21].

How the COVID-19 pandemic affected paediatric injuries in LMICs has been investigated to a limited extent [16, 18, 31], often through small-size studies focused on specific types of injuries (e.g., fractures, surgical emergencies) [32–34]. These few LMIC-based studies suggest that the impact on injury, in number and mechanism, may reflect the nature and rigidity of the restrictions implemented [35–40]. This is the case for studies from SSA where reductions in the volume of injured children presenting to EDs have been reported at the country level in Burkina Faso, Nigeria, and Zambia [34], and in the Western Cape, South Africa [41] while a persisting increase in help-seeking for intimate partner violence and sexual violence among adolescent girls and young women was reported in Nairobi, Kenya [42]. In Mozambique, a recent study revealed a 47% drop in the weekly number of hospital admission at the country’s four referral hospitals following the introduction of the first wave of restrictions in April 2020 [24], a drop that was mostly attributable to specific injury mechanisms (e.g., RTIs and falls). However, the drop was not long-lasting and, by the end of 2020 weekly numbers were back to the levels observed before the pandemic despite two consecutive waves of restrictions. The specificity of the two consecutive restrictions period at the country level has not been investigated, a question that could contribute to the knowledge gap on the broader question of changing injury volumes and patterns with changing restriction packages, with a focus on paediatric injuries. It could also increase the knowledge base on the characteristics of the clinical caseload in specialized hospitals.

## Methods

### Study design and setting

The analysis was based on a chart review of paediatric patients admitted with acute injuries to four regional referral hospitals in Mozambique. It was designed as a comparison of the injury characteristics during the restriction periods from 2020 with the same periods during 2019 the year before the pandemic. The first one, called the Emergency State (158 days), was between April 1^st^ and September 6^th^ and the second one, called the Public Calamity State (115 days), was from September 7^th^ to December 31^st^ [43]. The rationale for using two distinct periods following the introduction of the restrictions in the country is that the period from April 1^st^ to December 31^st^ is not homogeneous in the type of restrictions imposed. The Emergency State involved broad stay-at-home restrictions, schools and many workplaces being closed. During the Public Calamity State, some but not all schools remained closed, and most people returned to work. Restrictions were uniform across Mozambique during both periods and include the promotion of hand washing and disinfection of surfaces; enforcement of wearing face masks; social distancing, closing schools, churches, beaches, and gyms; reducing the number of workers; and instituting curfews and travel restrictions [15, 43].

The study encompassed the four regional referral hospitals, representing the highest level of care in Mozambique. In the Southern Region, the 1445-bed Hospital Central de Maputo is the country’s largest hospital. In the Central Region are the 1020-bed Hospital Central da Beira and 600-bed Hospital Central de Quelimane. In the Northern Region is the 549-bed Hospital Central de Nampula.

### Data sources and data collection

The data were comprised of patients aged 0–14 who were admitted to one of the included hospitals with acute injuries who required hospital-level care, defined as fulfilling one of the following inclusion criteria: boarding in the ED for at least 12 h, admission to a hospital ward, or admission to a paediatric ICU (PICU). The few patients who were admitted or were referred to the hospital later than 30 days after their initial injury were not considered acute and therefore excluded. We assumed that, as we are in the context of referral hospitals, there might be some delay between the time of the injury and the hospital visit/admission, e.g., due to the availability of an ambulance or the patient's conditions. The few cases with acute injury that were referred from other hospitals but admitted within up to 30 days of the injuries were thus included. In Mozambique, patients aged 15 years and over are not considered or treated as paediatric patients. Thus, only children aged 14 years and younger were included.

Data for this study were sourced from each hospital's ED records, hospital wards, and PICU and were collected by trained surgical residents and attending physicians. The case report form used was derived from the World Health Organization Injury Surveillance Guidelines [44] and included information on the injury site (hospital as a proxy of the area and setting for place of occurrence), the injured child (e.g., sex, age), the injury sustained (e.g., mechanism and nature of injury) the outcome (e.g., discharge, complications, death) and time (in the hospital and the PICU). Injury dates were determined according to the date of admission.

### Data analysis

For each restriction period, all hospitals aggregated and split the data by period and year, we took one variable at a time and compared the category-specific proportions in 2020 and 2019. The statistical significance of the differences between proportions was assessed using a t-test using a significance level of *p* < 0.05. The variables considered and their respective categories are indicated in Table 1 (site and child characteristics) and 2 (injury and outcome characteristics). We considered first the hospital (4 categories) and injury site (four categories: home, street/highway, leisure, and other) and two child characteristics: age (three categories) and sex (2 categories). Two injury characteristics were also studied (mechanism and nature) followed by four outcomes (discharged, complications, death and left before discharge). For the injury mechanism, intentional and unintentional injuries were considered separately, with four categories of unintentional injuries and one for intentional ones as the few cases reported were all resulting from interpersonal violence. Seven categories were used for the nature of the injury (fracture; burn; cut, bite, open wound; bruise or superficial injury; organ system injury; concussion; and others. In line with the WHO guidelines, we selected the most life-threatening injury multiple were present) and time at the hospital (total and PICU stay days).

**Table 1:** Differences in the number and proportion of cases by site and child characteristics stratified by restriction period, comparing the pre-pandemic to the pandemic year (2019 and 2020); t-test for differences in proportions

Site and child characteristics	Emergency state						Public Calamity State					
	2019		2020		∆ % 2020–2019		2019		2020		∆ % 2020–2019	
	n	%	n	%	∆	p	n	%	n	%	∆	p
**Hospital**												
Maputo	250	49.1	206	53.2	+ 4.1	0.193	242	56.0	148	40.9	-15.4	0.005
Beira	191	37.5	102	26.4	-11.1	0.040	131	30.3	137	37.9	+ 7.8	0.084
Quelimane	44	8.6	46	11.9	+ 3.3	0.326	25	5.8	29	8.0	+ 2.2	0.405
Nampula	24	4.7	33	8.5	+ 3.8	0.492	34	7.9	48	13.3	+ 5.4	0.225
**Setting**												
Home	332	65.2	280	72.4	+ 7.2	0.024	261	61.7	214	59.1	-2.6	0.279
Road	121	23.8	83	21.5	-2.3	0.334	119	28.1	93	25.7	-2.4	0.335
Leisure	38	7.5	11	2.8	-4.6	0.376	33	7.8	31	8.6	+ 0.8	0.467
Other	18	3.5	13	3.4	-0.1	0.379	10	2.4	24	6.6	+ 4.2	0.317
**Age (in years)**												
0–4	205	40.3	183	47.3	+ 7.0	0.078	158	36.6	116	32.0	-4.6	0.209
5–9	179	35.2	106	27.4	-7.8	0.093	175	40.5	159	43.9	+ 3.4	0.270
10–14	125	24.6	98	25.3	+ 0.7	0.457	99	22.9	87	24.0	+ 1.1	0.437
**Sex**												
Boys	331	65.0	252	65.1	+ 0.1	0.490	288	66.7	234	64.6	-2.1	0.305
Girls	178	35.0	135	34.9	-0.1	0.492	144	33.3	128	35.4	+ 2.1	0.365
**Total**	**509**	**100.0**	**387**	**100.0**			**432**	**100.0**	**362**	**100.0**		

The analyses were performed in STATA (STATA/SE 17.0) [44] and with an online *“P-value Calculator*” for differences in proportions and means [45].

## Results

Table 1 presents the distribution of the injuries by setting and child characteristic, restriction period for 2020 compared with the same period during the year before the restriction period. Additional columns provide the percent change by period, and the p-value of the t-test for comparison of proportions between years.

**Fig. 1:**
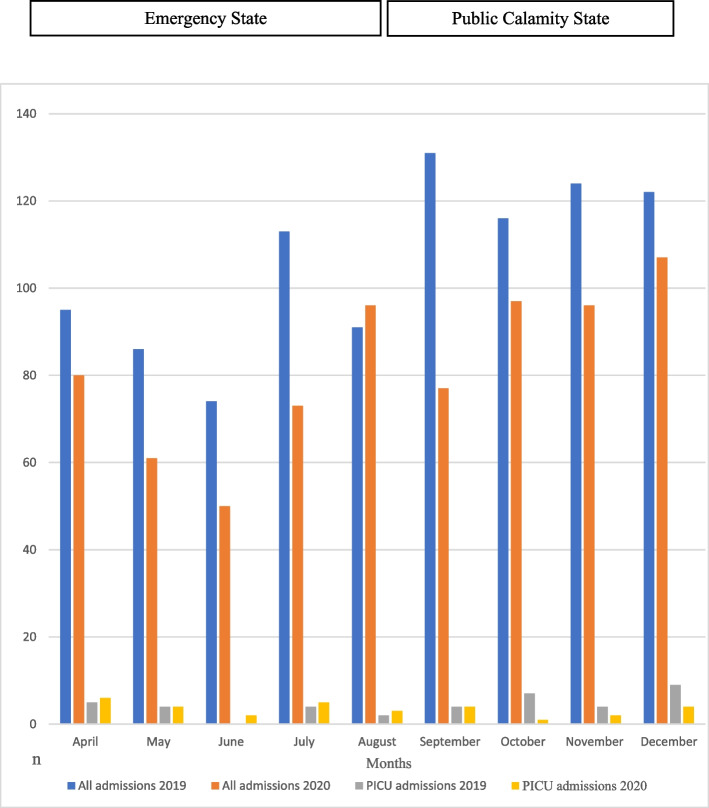
Monthly number of hospitalized pediatric injured patients overall and PICU admissions 2019–2020

For the same periods, the number of paediatric injuries admitted to the hospitals decreased during both restriction periods in 2020 compared to 2019: 509 to 387 (24.0%) during the Emergency State, and 432 to 362 (16.2%) during the Public Calamity State (from 941 to 749 (20.4%) for both periods aggregated). Figure 1 presents the monthly distribution of admissions at the hospital and the PICU for 2019 and 2020. It shows that there is a drop in admissions for each month of each period, except in August, the last month of the Emergency State and the most remarkable reduction is in July and September. The number of PICU admissions varies less and is more similar between years from April to September, than during the last three months when comparing 2019 to 2020.

The table shows that, as already known [24], not all hospitals registered a reduction in the number of injuries: Beira Central Hospital did not during the first restriction period and Maputo Central Hospital, did not during the second one. This translates into significant differences in proportions between years for those hospitals in the corresponding periods. Concerning the injury site, during the Emergency State the proportion (but not the number) of injuries that occurred at home significantly increased by 7% (*p* = 0.024) but none of the drops in proportions in the other setting was significant. Changes during the Public Calamity State were not significant. Likewise, there were no significant differences observed in the proportions of injuries by age category or sex despite period-specific differences in the direction of the differences.

Table 2 presents how the injury characteristics varied by restriction period, comparing 2020 to 2019. The total number of injuries reported as intentional was very low (*n* = 27) and none were reported in Quelimane central hospitals. Comparing the restrictions periods showed no differences in the Emergency State between years but a non-significant decrease of 3% in the Public Calamity State.

**Table 2:** Differences in the number and proportion of cases by injury and treatment characteristics stratified by restriction period, comparing the pre-pandemic to the pandemic year (2019 and 2020); t-test for differences in proportions and means

Injury characteristics and outcomes	Emergency state						Public Calamity State					
	2019		2020		∆ % 2020–2019		2019		2020		∆ % 2020–2019	
	n	%	n	%	∆	p	n	%	n	%	∆	p
**Mechanism**												
* Unintentional*												
Fall	192	37.7	123	31.8	-5.9	0.142	171	39.6	176	48.6	+ 9.0	0.039
Burns	177	34.8	179	46.3	+ 11.5	0.008	108	25.0	89	24.6	-0.4	0.470
Road traffic	106	20.8	61	15.8	-5.0	0.197	107	24.8	71	19.6	-5.2	0.197
Other	29	5.7	20	5.2	-0.5	0.350	30	7.0	24	6.7	-0.3	0.399
* Intentional*												
Interpersonal violence	5	1.0	4	1.0	-	-	16	3.7	2	0.6	-3.1	0.824
**Nature of injury**												
Fracture	237	46.8	149	38.5	-8.3	0.062	209	48.6	200	55.3	+ 6.7	0.083
Burns	177	34.8	179	46.3	+ 11.5	0.008	108	25.0	89	24.6	-0.4	0.470
Cut, bite, open wound	32	6.3	19	4.9	-1.4	0.165	25	5.8	20	5.5	-0.3	0.410
Bruise, superficial injury	23	4.5	14	3.6	-0.9	0.267	20	4.7	22	6.1	+ 1.4	0.184
Organ system injury	15	3.0	11	2.8	-0.2	0.435	25	5.8	13	3.6	-2.2	0.118
Concussion	8	1.6	9	2.3	+ 0.7	0.390	27	6.3	10	2.8	-3.5	0.089
Other	17	3.4	5	1.3	-2.1	0.192	18	4.2	8	2.2	-2.0	0.157
**Outcomes**												
Discharged	412	98.6	244	96.1	-2.5	0.035	325	96.7	285	96.3	-0.4	0.393
Complications	-	-	3	1.2	+ 1.2	-	6	1.8	7	2.4	+ 2.4	-
Death	2	0.5	2	0.8	+ 0.3	-	3	0.9	1	0.3	-0.6	-
Left before discharge	4	1.0	5	2.0	+ 1.0	-	2	0.6	3	1.0	+ 0.4	-
**Time** (avg days)	n	mean (sd)	n	mean (sd)	∆	p	n	mean (sd)	n	mean (sd)	∆	p
Hospital stays	505	11.7 (14.9)	430	12.2 (14.5)	+ 0.5	0.341	22	10.7 (13.3)	23	11.2 (14.2)	+ 0.5	0.342
PICU stay	384	21.0 (16.4)	361	19.9 (23.4)	-1.1	0.439	19	12.3 (10.6)	11	10.0 (5.5)	-2.3	0.203

For unintentional injuries, there are a few characteristics for which significant changes in proportions occurred, with some exceptions. During the Emergency State, there was an 11.5% increase in the percentage of burns (*p* = 0.008), a 5.9% no significant decrease in the percentage of falls, and an 8.2% decrease in the percentage of fractures. During the Public Calamity State, there was a 9.0% increase in falls (*p* = 0.039) and 6.7% no significant increase in fractures. In each period, there was a significant decrease in the percentage of patients injured that were discharged to the Emergency State. There was a notable decrease in RTIs (-5.0% and -5.2% respectively), but the differences in proportions as a mechanism of injury did not change significantly.

## Discussion

### Main findings

One first finding is that, besides expected [24] and period-specific reductions in the total number of injuries compared to the caseload of the previous year, the relative distribution of injuries across hospitals varied between restriction periods, showing a significant proportional increase of cases from the two larger hospitals: Beira Central Hospital during the first restriction period and Maputo Central hospital during the second. It is also of note that, when compared to the previous year, not all hospitals experienced reductions in the absolute number of injuries in both periods [24]. For instance, the number of injured children seen increased in the two smaller hospitals for each period, most remarkably in Nampula while the caseload increased at Beira Central Hospital during the second period. Turning to the injury pattern and the proportion of different injury, child and outcome attributes, the study reveals that very few significant changes occurred except for three in the first, most restrictive, period and one during the second one. In fact, during the Emergency State, the proportions of home injuries and burns were significantly higher than during the previous year (7.2% and 11.5% respectively) while that of discharged patients went down (by 2.5%). In that respect, it is of note that the only month where the number of cases was higher in 2020 than in 2019 was August (five cases), a result that we expect to be due to a peak in the increase of burns, those being more likely to occur in the home environment and August being part of the coldest month of the year, a month included in the Emergency State.

During the Public Calamity State, the absolute number of falls went up and their proportion rose significantly (by 9.0%). No additional significant differences in proportions were found for instance in the child age and sex distribution, in that of injury outcomes, or for the average time spent at the hospital for inpatients or patients admitted to the PICU.

That, compared to the same period the previous year, the total number of injuries dropped more during the first than the second restriction period may be explained by the nature of the restrictions which implied more confinement during the Emergency State, and a lower exposure to various sources of hazard, as suggested by reviews of the literature in the field [16, 18, 19]. This echoes the results from two American studies [38, 46] and one from Australia [47] which also considered two consecutive restriction periods and found this increased proportion only during the first phase [32]; it differs from a South African single-centre study [48] Further, that home-related injuries and burns increased in proportion (and even numerically for burns) during that same period can also be a consequence of the restrictions implying that children spent the greatest part of their time at home [18, 19] and that the risk for burns rose, most likely due to overcrowding [26–28]. Despite likely overcrowding, in our data there were no remarkable changes in the occurrence of violence-related injuries (can be related to the underreporting of the cases or fear to go to hospital due to COVID-19). This is in contrast with some [21] but not all [22] studies.

As the data show, the absolute number of RTIs went remarkably down during the Emergency State compared to the same period the year before. While the proportion of those injuries about the total number of injuries declined from 20.8% to 15.8%, the difference in proportions was not significant.

That we did not find any age- or sex-related differences in proportions compared to the previous year may be because all children were equally exposed to the restrictions. But there might also be differences that are not captured when comparisons are made with all injuries aggregated.

Once the restrictions were relaxed, there was an increase in the proportion of falls. This may reflect children being allowed to leave their homes and regain access to play and leisure activities outdoors. This increase can be further contextualized by seasonal injury variations in Mozambique. Injuries secondary to falls from height tend to peak in October and November, when coconut trees fruit [49], and in December when mango and citrus trees fruit. While under pre-pandemic conditions children would be spending this time at school, in 2020 school closures due to the pandemic meant children had more leisure/outdoor time. An increase in falls was also observed in other LMIC settings among both children and all age groups [32, 50], whereas results from HICs were mixed [19, 46].

### Implications of the study

This study reveals a change in the number of paediatric injuries treated in the country’s referral hospitals during the restriction periods imposed in the country in 2020. Whether this reflects the true burden or of a reduced likelihood to seek care remains to be determined. It is also unclear whether those fewer injury victims received adequate care after presentation or admission at the hospital given the pressed health care environment where they were treated.

For their part, the significant changes in the relative importance of some injury characteristics may be attributed to exposure differentials induced by some of the restrictions: for example, school closures had children spending time in parks for leisure activities. This is alongside more crowded home environments due to lockdown restrictions increasing the risk of injury [51–53].

On the treatment side, hospital administrators and healthcare workers involved in the treatment of childhood injuries will be better prepared to provide optimal care with a stronger understanding of these injury patterns.

### Strengths and limitations

One strength of this study is its countrywide coverage encompassing all Mozambican central hospitals, allowing for an overall picture of the situation in the country. It also covers the whole period of restrictions in 2020, allowing for an exploration into what happened during each of the two restriction periods. A comparison with the same period before the pandemic also has the advantage to avoid comparing with a period when the infection begins to spread globally and in the country, and people change their lifestyles and behaviour. It also accounts for seasonal variations that can be masked when compared with preceding months.

Limitations exist due to the use of secondary data collected from paper patient charts, stored in the records of the hospital's ED. In the worst case, patient files may have been lost and critical information went missing or cases completely disappeared. As there is no reason to expect any systematic bias in that respect, the relative differences observed countrywide may not be seriously influenced. However, conditions at two hospitals need to be underlined: a fire at Maputo Central Hospital in mid-2019 and a flood in December 2019 in Beira [54]. Some of the consequences of the fire in the archive include inevitably a loss of both files for the first consultation and a loss of follow-up information (leading to under-reporting and missing case information). Some of this could be counteracted thanks to the emergency service and the logbooks at the wards having basic information. By destroying the roads and limiting access to the health facilities, the flood and cyclone in Beira impeded healthcare seeking and disrupted the provision of care to the extent that the usual daily patient numbers in primary health facilities dropped up to 300 per cent to about 20–25. Both situations could have led to an underestimation of the number of cases admitted in 2019, and consequently, an underestimation of the reduction of the number of cases before and during the restriction periods.

## Conclusion

During pandemic restrictions implemented in Mozambique in 2020, each restriction period saw a drop in the volume of injury admissions at central hospitals, but the pattern of child, injury and outcome characteristics did not change much, except for an excess of home and burn injuries in the first, more restrictive period. Whether this reflects the nature of the restrictions only or other individual or health systems related mechanisms remains to be determined. Future studies on quality injury care may also reveal whether the injured children received similar care during the restriction periods compared to before. Altogether, the results may inform healthcare services in their future response to injury and trauma care during periods of the pandemic.

## Data Availability

All data generated or analysed during the current study are included in this published article and its supplementary information files.

## Abbreviations

LMIC: Low-Middle Income Country
HIC: High-Income Country
ED: Emergency Department
SSA: Sub-Sahara Africa
PICU: Paediatric Intensive Care Unit
RTI: Road Traffic Injury
AFTCOR: Africa Taskforce on Coronavirus Preparedness and Response
